# Bacterial foodborne illness and *Escherichia coli* O157:H7 strain infection among asymptomatic food handlers in Northeast Ethiopia: Implication for hygienic practices and mass‐screening

**DOI:** 10.1002/hsr2.2199

**Published:** 2024-06-18

**Authors:** Kalu Adefrash, Bekele Sharew, Wubalem Amare, Agumas Shibabaw

**Affiliations:** ^1^ Laboratory and Diagnostic Services Unit Shiwa Robit Primary Hospital Shewa Robit Ethiopia; ^2^ Department of Medical Laboratory Sciences, College of Medicine and Health Sciences Debre Tabor University Debre Tabor Ethiopia; ^3^ Department of Chemical Engineering, School of Mechanical and Chemical Engineering, Kombolcha Institute of Technology Wollo University Kombolcha Ethiopia; ^4^ Department of Medical Laboratory Sciences (Medical Microbiology Unit), College of Medicine and Health Sciences Wollo University Dessie Ethiopia

**Keywords:** bacterial infection, drug resistance patterns, food‐borne illness, food establishment, food handlers

## Abstract

**Background and Aims:**

Food‐borne illness is a public health concern in developing countries because of improper food handling and sanitation practices, irregular medical checkups, lack of clean water supplies, and inadequate education among food handlers. This study investigated the burden of bacterial food‐borne illness, antibiotic resistance patterns, and associated factors among food handlers in prison and nonprison food establishment settings.

**Methods:**

A cross‐sectional study was conducted from August 2022 to January 2023 among asymptomatic food handlers in Shewa Robit town. A total of 384 food handlers participated. Data were collected using structured questionnaires. Stool and hand swab samples were collected and cultivated onto MacConkey agar, xylose‐lysine‐deoxycholate, Mannitol salt agar, and blood agar, and incubated at 37°C. Bacterial species were identified using biochemical tests and gram staining. Mueller–Hinton agar was used in Kirby Bauer's disk diffusion method. Data were entered and analyzed using SPSS. Descriptive and logistic regression analysis were performed.

**Results:**

Fecal and hand carriage rate of bacterial isolates were 106 (27.6%), and 214 (55.7%), respectively. Out of the 102 bacterial isolates, the most common ones from stool samples were *Escherichia coli* 71 (18.5%), *Klebsiella aerogenes* 12 (3.1%), and *Salmonella* spp. 7 (1.8%). Among 214 bacterial isolates, coagulase‐negative *Staphylococci* 115 (29.9%) and *Staphylococci aureus* 66 (17.3%) were identified from hand swab samples. Hand washing practice after restroom with water (adjusted odds ratio [AOR] = 2; 95% confidence interval [CI]: 1.16–3.45), irregular medical checkups (AOR = 2.49; 95% CI: 1.35–4.59), and did not receive food safety and hygiene training (AOR = 2.33; 95% CI: 1.34–4.05) were statistically significant association with food‐borne illness.

**Conclusions:**

Foodborne pathogens pose a serious health risk in the study areas. The level of antimicrobial resistance are also concerning. Food handlers should therefore get strict regular health education, medical checkups, and training programs to prevent the spread of infections to the customers.

## 1 INTRODUCTION

Food‐borne illness constitute a significant public health concern worldwide. Food‐borne illness claim the lives of two million people annually in developing countries.^1^ Food‐borne illness are more common in low‐ and middle‐income countries because food safety is rarely given legislative attentions.^2^

Inadequate food handling and sanitation practices, a lack of clean water, poverty, and inadequate training for food handlers are among the factors contributing to food‐borne illness as a public health concern in developing countries.^3^ Harmful substances and pathogenic microorganisms may contaminate food during food production, transportation, preparation, storage, and servicing. Certain people have been known to be impacted by food contamination caused by pathogenic organisms.^4^

Food‐borne bacteria can spread through contaminated food, water, fingernails, and other materials, both directly and indirectly. This implies that one potential means of spreading those bacteria among humans by fecal‐oral transmission.^5^ Food handlers within a community have a big role in the spread of food‐borne illness.^6^ The asymptomatic nature of food‐borne bacteria makes it challenging to prevent and control food‐borne infections.^7^

Humans are incredibly active and productive when they consume healthy and safe food. A variety of bacterial species can cause foodborne illness when people consumed contaminated vegetables, fruits, meats, and dairy products. The most vulnerable groups to these infections are children and those with immuno‐compromised individuals. Conversely, the most common bacteria found in food handlers are *Escherichia coli*, *Salmonella* spp*., Shigella* spp*., Clostridium* spp*., Campylobacter* spp., and *S. aureus*.^8^ Despite the fact that the majority of *E. coli* strains are not harmful, some, like *O157: H7* strain, carry the exotoxin that can seriously poison humans who eat certain foods.^9^

*E. coli* O157:H7 is one of the pathogenic serotype associated with severe diarrheal diseases that lead to hemorrhagic colitis, hemolytic uremic syndrome, and death, especially in immunocompromised individuals. The source of this bacterium is mainly food and animal foods in which the pathogen seeps into the food chain from improper hygienic practices either during the slaughtering of the animal or during food processing and servicing.^10^ One study from food of bovine origin in Ethiopia reported that the prevalence rates of *E. coli* O157:H7 was 6.5%.^11^

According to various reports, antimicrobial resistance (AMR) poses a serious threat to public health everywhere,^12^ including in Ethiopia with different AMR reports among humans and animal products.^11^, ^13^, ^14^ Human can contract resistant bacteria from a variety of sources such as food products, the environment, and food handlers. A significant issue in developing countries is the emergence and spread of antibiotic‐resistant bacteria, which can be easily contracted as a result of substandard living conditions and limited access to medical care.^15^

The frequency of food‐borne illness, and *E. coli* O157:H7 infection distribution can be attributed due to variations on food production methods, inadequate food storage facilities, unsanitary food handling practices, resource constraints, and inadequate enforcement of regulatory norms.^16^ Few previous studies in Ethiopia focused on selected bacterial species such as *Salmonella* and *Shigella*, and intestinal parasites distribution among food handlers instead of all the bacterial pathogens causing food‐borne illness. Therefore, this study investigated the prevalence of food‐borne illness, *E. coli* O157:H7 strains, antibiotic resistance patterns, and associated factors among asymptomatic food handlers in Northeast Ethiopia.

## 2 METHODS AND MATERIALS

### 2.1 Study design, period, and settings

A cross‐sectional study was carried out in both prison and nonprison settings in Shewa Robit town between August, 2022 and January, 2023. The Shewa Robit town is located in North Shewa Zone, Amhara Regional State, and 220 km from the capital city, Addis Ababa. An estimated of 54,307 people are living in the town according to the 2007 Central Statistical Agency census data. A total of 145 food handlers were employed in. Based on the Shewa Robit culture and Tourism Bureau information, there were a total of 16 hotels (with 211 food handlers), seven cafes (with 79 hand handlers), and 16 restaurants (with 90 food handlers). A source population consisted of all food handlers who were employed in Shewa Robit prison and nonprison settings. Food handlers who were actively involved in the preparation, handling, processing, or serving of food in selected nonprison settings and Shewa Robit Federal prison during the study period, as a study population.

### 2.2 Sample size and sampling technique

Using a single population proportion formula, the final sample size was determined by taking into account 50% prevalence, 5% margin of error (*d* = 0.05), and 95% confidence interval (*z* = 1.96); *n* = (Zα/2) ^2^ *p*(1‐*p*)/*d* ^2^. Thus, 384 study participants were recruited and participated in this study. Food establishment setting including 106 food handlers from prison setting, 66 from restaurants, 154 from hotels, and 58 from café. Food handlers were selected using a simple random sampling (lottery method) technique in each study setting.

### 2.3 Inclusions and exclusions criteria

All selected asymptomatic food handlers who were working in prison setting, hotels, cafés, and restaurants in Shewa Robit town were included in the study. Food handlers with clinical symptoms such as headache, nausea, vomiting, coughing, and diarrhea, and those who took antibiotics for 2 weeks before the study were excluded from the study.

### 2.4 Demographic and hygienic practices data collection

Structured questionnaires comprising demographic and related factors (eight independent variables) and food hygiene practices (13 independent variables) data were collected. The questionnaires were pretested and validated before the actual data collection. The independent variables were socio‐demographic information such as age, sex, marital status, level of education, monthly income, job position, service year, and hand washing habits before preparing food, hand washing after restroom with soap or water, cleaning equipment, fingernail status, training experience, status of medical checkups, wearing an apron and hair covering, and knowledge of food‐borne illnesses. Selected food handlers were interviewed face‐to‐face by trained nurses. Continuous supervision, monitoring, and daily checks for data completeness were used to guarantee data quality. Data collectors were trained on how to collect data and samples from study participants.

### 2.5 Samples collection, transportation, and processing

Using a clean, dry, and leak‐proof stool cup, 2 g of stool samples were taken from each food handler. The specimens were put into the Cary‐Blair transport medium, and transported to Debre Birhan compressive specialized hospital laboratory for laboratory analysis. Additionally, sterile cotton‐tipped swabs dampened with normal saline were used to collect hand swabs from the palms of both hands of each food handler, and Amiens transport media was used to transport the samples.^17^

### 2.6 Bacterial culture, isolation, and identification

Wire loop was used to streak samples or microbial cultures onto the surface of solid culture media plates. Samples were inoculated on culture media using zig‐zag streaking technique (quadrant streak plating) to identify pure colony. Using a sterile wire loop, a stool sample was directly inoculated onto xylose‐lysine‐deoxycholate (XLD) and MacConkey (MAC) agar plates. The inoculated culture media was incubated at 37°C for 18–24 h, and examined for bacterial growth. The bacterial growth was detected and identified by colonial morphology and pigmentation, Simmons citrate, indole formation oxidase test, H_2_S production, urease production, and citrate utilization.^18^ Additionally, the swab sample was cultivated onto blood agar plate (BAP), MAC, and XLD, and Mannitol salt agar, and incubated at 37°C for 18–24 h. Standard biochemical tests including indole synthesis, Simmons citrate, urease, motility test, lysine, gas and sulfur production, glucose, and lactose fermentation, were used to detect and identify the bacterial species.^19^

### 2.7 Identification of *E. coli* O157:H7 strain

The *E. coli* strain*s* were differentiated using Sorbitol MacConkey Agar. When *E. coli* O157:H7 was grown on MAC with Sorbitol and incubated aerobically at 37°C for 18–24 h.

### 2.8 Antimicrobial susceptibility testing

The Kirby–Bauer disk diffusion method was used to test for antibiotic susceptibility in accordance with Clinical Laboratory Standard Institute (CLSI) guidelines. Freshly produced bacteria were suspended in 3–5 mL of normal saline to create bacterial suspensions, and the turbidity was then adjusted to meet the 0.5 McFarland standard. A sterile cotton swab was dipped and rotated several times and placed up against the test tube wall. Mueller Hinton agar plate was then completely swabbed, and antimicrobial disks were placed on top of the surface. The following antimicrobial agents were used for bacterial isolates: amoxicillin‐clavulanic acid (30 μg), cefotaxime (5 μg), ceftazidime (10 μg), ceftriaxone (30 μg), chloramphenicol (30 μg), ciprofloxacin (5 μg), trimethoprim‐sulfamethoxazole (1.25/23.75 μg), amikacin (30 μg), cefepime (30 μg), meropenem (10 μg), gentamicin (10 μg), tobramycin (10 μg), erythromycin (15 μg), and oxacillin (30 μg). The CLSI 2020 guideline was utilized to interpret the resistance and sensitivity.^20^ Reference strains such as *Pseudomonas aeruginosa* (ATCC 27853), *E. coli* (ATCC 25922), and *Staphylococcus aureus* (ATCC 29213) were used to assess the quality of the culture media and potency of antibiotic disks.

### 2.9 Data management and analysis

Data were entered into Epidata software and exported into SPSS version 26 for analysis. Descriptive statistics and logistic regression analyses were used. Model fitness was checked using the Hosmer–Lemeshow test. Bivariable logistic regression was computed and variables with a *p*‐value ≤ 0.2 were entered into the multivariable logistic regression analysis to control confounding variables. The crude and adjusted odds ratio were computed and *p*‐value < 0.05 was considered statistically significant.

### 2.10 OPERATIONAL DEFINITIONS

Food handler: is a person who directly engages in the preparation, processing, handling, and serving of food in the food establishment settings.
Nonprison setting: Food facility settings such as hotels, restaurants, and café in Shewa Robit town but not include prison setting.
Multiple drug resistance (MDR): Non‐susceptibility to at least one or more antimicrobial agent in three or more antimicrobial categories.

### 2.11 Ethical approval and patient consent

Ethical approval was obtained from the Research and Ethics Review Committee of College of Medicine and Health Sciences, Wollo University (Ref: CMHS/12/2022). A formal written letter was obtained from the Shewa Robit city administration health office and the prison authorities. Written informed consent was obtained from each study participant before data collection and participated voluntarily. The purpose and procedures of the study were explained to each study participant during data collection. Positive results were communicated to the study participant, prison health officials, and Shewa Robit city administration health office for further assessment and treatment. The confidentiality was maintained.

## 3 RESULTS

### 3.1 Socio‐demographic characteristics of study participants

A total of 384 study participants were enrolled in the study. The study participants ranged in age from 16 to 42 years old, with a mean age of 24 years (SD ± 4.5). Among 384 study participants, the majority of study participants including 289 (75.3%) females, 252 (65.6%) waiters, 231 (60.2%) single participants, and 232 (60.4%) enrolled in the primary school (Table 1).

**Table 1:** Socio‐demographic characteristics of food handlers in prison and nonprison food establishment settings in Shewa Robit town (*N* = 384).

Characteristics	Category	Frequency (*n*, %)
Gender	Male	95 (24.7)
	Female	289 (75.3)
Age	16–24	226 (58.9)
	25–34	108 (28.1)
	>35	50 (13)
Marital status	Single	231 (60.2)
	Married	142 (36.9)
	Divorced	11 (2.9)
Monthly income	<1000	23 (6)
	1000–2000	335 (87.2)
	>2000	26 (6.8)
Service years	1–2	99 (25.8)
	>2	285 (74.2)
Educational status	No formal education	51 (13.3)
	Elementary school	232 (60.4)
	Secondary school	98 (25.5)
	Collage and above	3 (0.8)
Job position	Cooker	127 (33.1)
	Waiter	252 (65.6)
	Mixed	5 (1.3)
Food establishment settings	Prison	106 (27.6)
	Hotel	154 (40.1)
	Restaurant	66 (17.2)
	Café	58 (15.1)

### 3.2 Hygiene practice of food handlers

Many food handlers stated that they wash their hands with water alone (66.5%). Three hundred twenty‐eight (85.4%) and 346 (90.1%) food handlers reported that they always wash their hands before preparing food and after using restroom, respectively. But, 141 (36.7%) of them made it a routine to wash their hands after handling unclean objects. Out of the total participants, only five (1.3%) of the participants have a certificate in food safety and hygiene. A total of 112 (29.2%) food handlers received in‐service training on safe food preparation and handling practices (Table 2).

**Table 2:** Food hygiene practices of food handlers at prison and nonprison food establishment settings in Shewa Robit town (*N* = 384).

Hygiene practices and trainings	Category	Frequency (*n*, %)
Regular medical checkups	Yes	89 (23.2)
	No	295 (76.8)
Certificate in food safety and hygiene	Yes	5 (1.3)
	No	379 (98.7)
Trained in food hygiene and safety	Yes	112 (29.2)
	No	372 (70.8)
Hand washing practice before handling food	Yes	328 (85.4)
	No	56 (14.6)
Practice of trimming a fingernail	Trimmed	349 (90.9)
	Not trimmed	35 (9.1)
Habit of wearing a clean apron	Yes	239 (62.2)
	No	145 (37.8)
Habit of wearing a clean hair garment	Yes	111 (28.9)
	No	273 (71.1)
Hand washing practices after restroom	Yes	346 (90.1)
	No	38 (9.9)
Hand washing practices after restroom with	Soap	129 (37.3)
	Water alone	217 (62.7)
Had separate cooking and cleaning areas	Yes	282 (73.4)
	No	102 (26.6)
Hand washing practices after touching dirty material	Yes	141 (36.7)
	No	243 (63.3)
Knowledge about food‐borne diseases	Yes	245 (63.8)
	No	139 (36.2)
Food waste disposal areas	Dust bin	179 (46.6)
	Open waste area	205 (53.4)

### 3.3 Hand carriage rate of bacteria

**Figure 1:**
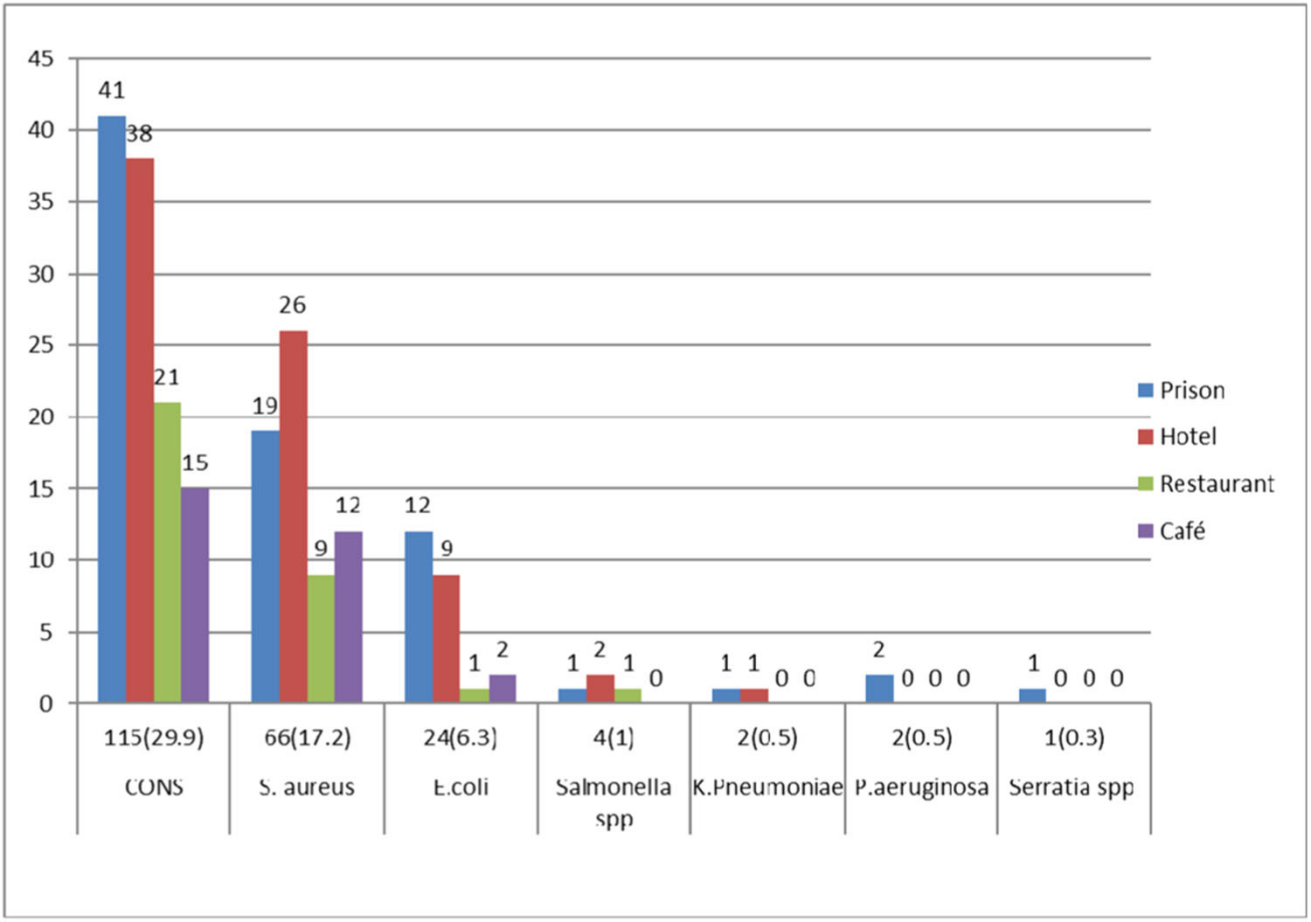
Hand carriage rate of bacterial contamination among food handlers in prison and nonprison food establishment settings in Shewa Robit town.

Using hand swabs, 214 different bacteria were identified. Of these, 77 bacterial isolates were detected among food handlers in the prison setting. Among these, coagulase‐negative *Staphylococci* were the predominant bacteria 115 (29.9%) followed by *S. aureus* 66 (17.2%), *E. coli* 24 (6.3%), and *Salmonella* species 4 (1%) (Figure 1).

### 3.4 Fecal carriage rate of bacteria

**Figure 2:**
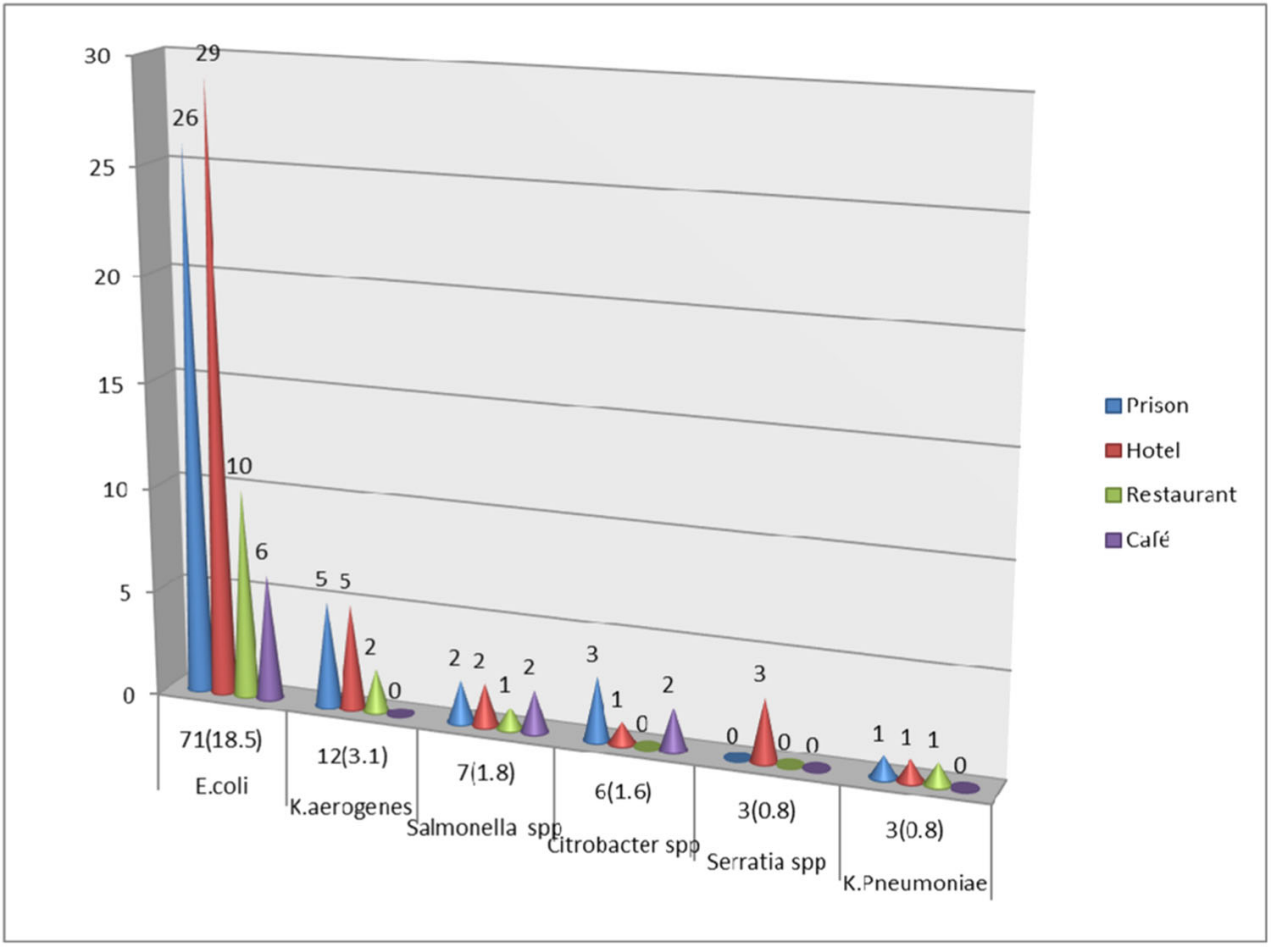
Fecal carriage rate of bacterial species among food handlers at prison and nonprison food establishment settings in Shewa Robit town.

In stool culture, 102 different bacteria were identified. Of these, 37 bacterial isolates were detected among food handlers in the prison setting. *E. coli* 71 (18.5%), *K. aerogenes* 12 (3.1%), *Salmonella* 7 (1.8%) and *Citrobacter* species 6 (1.6%) were the most common bacterial isolates (Figure 2).

### 3.5 AMR pattern of bacterial isolates

Within the bacterial isolates, *S. aureus* from hand swabs exhibited the highest resistance patterns to erythromycin (66.2%), trimethoprim‐sulfamethoxazole (57.6%), and ciprofloxacin (49.9%). Sixty‐eight percent of *S. aureus* were susceptible to oxacillin (Table 3).

**Table 3:** Antimicrobial resistance patterns of bacterial isolates detected from hand swabs among food handlers at prison and nonprison food establishment settings in Shewa Robit town (*N* = 384).

Antibiotic	AST	Bacterial isolates						
		*CoNS*	*Staphylococcus aureus*	*Escherichia coli*	*Klebsiella pneumoniae*	*Serratia*	*Salmonella*	*Pseudomonas aeruginosa*
CTR	S	‐	‐	13 (54.2)	2 (100)	‐	3 (75)	‐
	R	‐	‐	11 (45.8)	‐	1 (100)	1 (25)	‐
C	S	66 (57.3)	45 (68.1)	17 (70.8)	2 (100)	‐	4 (100)	‐
	R	49 (42.7)	21 (31.9)	7 (32.2)	‐	1 (100)	‐	
CAZ	S	‐	‐	22 (91.7)	2 (100)	1 (100)	4 (100)	1 (50)
	R	‐	‐	2 (8.3)	‐	‐	‐	1 (50)
GM	S	51 (44.3)	37 (56.1)	13 (54.2)	2 (100)	1 (100)	3 (75)	‐
	R	64 (55.7)	29 (43.9)	11 (45.8)	‐	‐	1 (25)	2 (100)
FEP	S	‐	‐	21 (87.5)	2 (100)	1(100)	4 (100)	‐
	R	‐	‐	3 (12.5.)	‐	‐	‐	‐
CTX	S	‐	‐	10 (41.7)	1 (50)	‐	4 (100)	‐
	R	‐	‐	14 (58.3)	1 (50)	1 (100)	‐	‐
CIP	S	52 (45.2)	37 (56.1)	13 (54.2)	2 (100)	1 (100)	2 (50)	‐
	R	63 (54.8)	29 (43.9)	11 (45.8)	‐	‐	2 (50)	2 (100)
SXT	S	43 (37.4)	28 (42.4)	9 (37.5)	2 (100)	‐	3 (75)	1 (50)
	R	72 (62.6)	38 (57.6)	15 (62.5)	‐	1(100)	1 (25)	1 (50)
OX	S	72 (62.6)	45 (68.1)	‐	‐	‐	‐	‐
	R	43 (37.3)	21 (31.9)	‐	‐	‐	‐	‐
E	S	37 (32.2)	23 (34.8)	‐	‐	‐	‐	‐
	R	78 (67.8)	43 (65.2)	‐	‐	‐	‐	‐
MER	S	‐	‐	23 (95.8)	1 (50)	‐	4 (100)	2 (100)
	R	‐	‐	1 (4.2)	1 (50)	1 (100)	‐	‐
AM	S	‐	‐	20 (83.3)		1 (100)	4 (100)	‐
	R	‐	‐	4 (16.7)		‐	‐	‐
AMC	S	‐	‐	6 (25)	1(50)	1(100)	3 (75)	‐
	R	‐	‐	18 (75)	1(50)	‐	1 (25)	‐
TOB	S	‐	‐	‐	‐	‐	‐	2 (100)
	R	‐	‐	‐	‐	‐	‐	‐

Abbreviations: AM, amikacin; AMC, amoxicillin–clavulanic acid; AST, antimicrobial susceptibility testing; C, chloramphenicol; CAZ, ceftazidime; CFR, ceftriaxone; CIP, ciprofloxacin; CTX, cefotaxime; E, erytromicin; FEP, Cefepime; GM, gentamycin; MER, meropenem; OX, oxacillin; SXT, cotrimoxazole; TOB, tobramycine.

*E. coli* from a stool sample was more susceptible to meropenem (92.9%), cefepime (87.3%), cefotaxime (85.9%), and amikacin (83.1%). A 100% sensitivity to meropenem, cefepime, cefotaxime, and ceftazidime was seen in *Salmonella* species from fecal samples (Table 4).

**Table 4:** Antimicrobial resistance patterns of fecal‐bacterial isolates among food handlers at prison and nonprison food establishment settings in Shewa Robit town.

Antibiotics	AST	Bacterial isolates					
		*Escherichia coli*	*Citrobacter*	*Klebsiella pneumonia*	*Serratia*	*Salmonella*	*Klebsiella aerogenes*
CFR	S	46 (64.7)	5 (83.3)	2 (66.7)	3 (100)	5 (71.4)	7 (58.3)
	R	25 (35.3)	1 (16.7)	1 (33.3)	‐	2 (28.6)	5 (41.7)
C	S	49 (69.1)	6 (100)	3 (100)	2 (66.7)	3 (42.9)	8 (66.7)
	R	22 (30.9)	‐	‐	1 (33.3)	4 (57.2)	4 (33.3)
CAZ	S	56 (78.8)	4 (66.7)	‐	2 (66.7)	7 (100)	9 (75)
	R	15 (21.1)	2 (33.3)	3 (100)	1 (33.3)	‐	3 (25)
GM	S	49 (69.1)	2 (33.3)	3 (100)	2 (66.7)	3 (42.9)	4 (33.3)
	R	22 (30.9)	4 (66.7)	‐	1 (33.3)	4 (57.1)	8 (66.7)
FEP	S	62 (87.3)	6 (100)	3 (100)	3 (100)	7 (100)	10 (83.3)
	R	9 (12.6)	‐	‐	‐	‐	2 (16.2)
CTX	S	61 (85.9)	6 (100)	3 (100)	3 (100)	7 (100)	10 (83.3)
	R	10 (14.1)	‐	‐	‐	‐	2 (16.7)
CIP	S	42 (59.1)	1 (16.7)	1 (33.3)	2 (66.7)	4 (57.1)	5 (41.7)
	R	29 (40.8)	5 (83.4)	2 (66.6)	1 (33.3)	3 (42.9)	7 (58.3)
SXT	S	28 (39.4)	5 (83.3)	2 (66.7)	1 (33.3)	3 (42.9)	5 (41.7)
	R	43 (60.5)	1 (16.7)	1 (33.3)	2 (66.7)	4 (57.1)	7 (58.3)
MER	S	66 (92.9)	6 (100)	2 (66.7)	3 (100)	7 (100)	12 (100)
	R	5 (7.1)	‐		‐	‐	‐
AM	S	59 (83.1)	6 (100)	1 (33.3)	3 (100)	6 (85.7)	‐
	R	12 (16.9)	‐	2 (66.7)	‐	‐	‐
AMC	S	46 (64.7)	5 (83.3)	3 (100)	2 (66.7)	6 (85.7)	‐
	R	25 (35.3)	1 (16.7)	‐	1 (33.3)	1 (14.3)	‐

Abbreviations: AM, amikacin; AMC, amoxicillin–clavulanic acid; AST, antimicrobial susceptibility testing; C, Chloramphenicol; CAZ, ceftazidime; CFR, ceftriaxone; CIP, ciprofloxacin; CTX, cefotaxime; FEP, cefepime; GM, gentamycin; MER, meropenem; SXT, cotrimoxazole.

### 3.6 MDR pattern of bacterial isolates

The overall MDR rate of this study was 17.9% (316/177). Of the detected bacterial isolates from fecal and hand samples, 58 (56.9%) and 121 (56.5%) bacteria had MDR levels. *Salmonella* species and *K. aerogenes* showed comparatively higher rate of MDR, with average resistance of 71.4% and 91.6%, respectively (Tables 5 and 6).

**Table 5:** Multidrug resistance (MDR) patterns of fecal bacterial isolates detected from food handlers at prison and nonprison food establishment settings in Shewa Robit town.

Level of antibiotic resistance (*n*, %)							
Bacteria isolates	R0	R1	R2	R3	R4	>R5	>R3 (MDR)
*Escherichia coli* (71)	9 (12.6)	12 (16.9)	14 (19.7)	19 (26.7)	10 (14)	7 (9.8)	36 (50.7)
*Klebsiella pneumonia* (3)	0 (0)	0 (0)	1 (33.3)	1 (33.3)	1 (33.3)	0 (0)	2 (66.6)
*Citrobacter* spp.(6)	0 (0)	1 (16.6)	3 (50)	2 (33.3)	0 (0)	0 (0)	2 (33.3)
*Klebsiella aerogenes* (12)	0 (0)	0 (0)	1 (8.3)	6 (50)	4 (33.3)	1 (8.3)	11 (91.6)
*Serratia* spp. (3)	1 (33.3)	0 (0)	0 (0)	1 (33.3)	1 (33.3)	0 (0)	2 (66.6)
*Salmonella* (7)	1 (14.2)	1 (14.2)	0 (0)	5 (71.4)	0 (0)	0 (0)	5 (71.4)
Total (*n* = 102)	11 (10.7)	14 (13.7)	19 (18.6)	34 (33.3)	16 (15.6)	8 (7.8)	58 (56.8)

*Note*: R0: resistance to no antibiotics, R1–5: resistance to 1, 2, 3, 4, and 5 antibiotics; ≥R3: resistance to three or more antibiotics from different classes.

**Table 6:** Multidrug resistance (MDR) patterns of bacterial isolates detected from hands of food handlers at prison and nonprison food establishment settings in Shewa Robit town.

Level of antibiotic resistance *n* (%)							
Bacterial isolates	R0	R1	R2	R3	R4	>R5	>R3 MDR
*Staphylococcus aureus* (66)	9 (13.6)	5 (7.5)	18 (27.2)	16 (24.2)	15 (22.7)	3 (4.5)	34 (16)
CoNS (115)	11 (9.5)	16 (13.9)	26 (22.6)	28 (24.3)	19 (16.5)	15 (13)	62 (29.2)
*Escherichia coli* (24)	2 (8.3)	1(4.1)	1 (4.1)	8 (33.3)	6 (25)	6 (25)	20 (9.4)
*Klebsiella pneumonia* (2)	0	0	1 (50)	0	1 (50)	0(0)	1 (0.5)
*Serratia* spp.(1)	0	0	0	0	0	1 (100)	1 (0.5)
*Salmonella* spp. (4)	1 (25)	1 (25)	1 (25)	0	1 (25)	0 (0)	1 (0.5)
*Pseudomonas aeruginosa* (2)	0	0	0	2 (100)	0	0	2 (100)
Total (*n* = 214)	23 (10.8	23 (10.8)	47 (22.1)	54 (25.2)	42 (19.8)	25 (11.7)	121 (56.5)

*Note*: R0: resistance to no antibiotics, R1–5: resistance to 1, 2, 3, 4, and 5 antibiotics; ≥R3: resistance to three or more antibiotics from at least three or more different classes.

### 3.7 Prevalence and AMR patterns of EHEC O157:H7

There were four enterohemorrhagic *E. coli* strains (4.2%, 4/95) which were O157:H7 serotypes. A half percentage (50%) of *E. coli* O157:H7 strains were resistant to ceftriaxone, trimethoprim‐sulfamethoxazole, and chloramphenicol. Amoxacillin‐clavulanic acid, meropenem, cefepime, amikacin, gentamycin, ciprofloxacin, ceftazidime, ciprofloxacin, and cefotaxime showed 100% efficacy against the strains.

### 3.8 Associated factors with hand carriage of bacterial isolates

Out of all the associated factors evaluated in this study, hand washing practices of food handlers after restroom just water had a significant association (adjusted odds ratio [AOR] = 2.03, 95% confidence interval [CI]: 1.26–3.27, *p* < 0.001) with hand bacterial colonization compared to the counterpart (Table 7).

**Table 7:** Associated factors for acquisition of bacterial hand colonization using bivariate and multivariate analysis among food handlers in prison and nonprison food establishment settings in Shewa Robit town.

Variables	Category	Bacterial colonization		COR (95% confidence interval [CI])	*p*‐Value	Adjusted odds ratio (95% CI)	*p*‐Value
		Yes	No				
Habit of wearing hair garment	Yes	69	42	Ref			
	No	145	128	1.53 (0.99–2.42)	0.05	1.2 (0.71–4.31)	0.47
Hand washing practices after restroom	Soap	79	50	Ref			
	Water alone	92	125	2.13 (1.51–3.32)	0.001	2.03 (1.26–3.27)	0.004*
Hand washing practice after touching dirty material	Yes	58	83	Ref			
	No	130	113	0.60 (0.39–0.92)	0.02	2.06 (1.28–3.31)	0.30
Regular medical checkup	Yes	58	31	Ref			
	No	156	139	1.64 (1.01–2.65)	0.04	1.55 (0.88–2.75)	0.12
Food facility settings	Prison	66	40	2.78 (1.13–4.17)	0.019	2.04 (0.9–4.31)	0.06
	Hotel	66	88	0.99 (0.53–1.82)	0.97	2.04 (0.096–0.43)	0.061
	Restaurant	31	35	1.16 (0.57–2.37)	0.66	1.07 (0.54–2.10)	0.83
	Café	25	33	Ref			

Abbreviation: COR, crude odds ratio.

*Significant variables at *p* < 0.05.

### 3.9 Associated factors with fecal carriage bacteria isolates

Different factors were assessed for possible association with fecal carriage among food handlers. Of these checked associated factors, irregular medical checkups (AOR = 2.49, 95% CI: 1.35–4.59, *p* < 0.001), did not receive food safety and hygiene training (AOR = 2.33, 95% CI: 1.34–4.05, *p* < 0.001), and hand washing practice after restroom with just water (AOR = 2, 95% CI: 1.16–3.45, *p* < 0.001) were significantly associated with fecal bacterial colonization (Table 8).

**Table 8:** Bivariate and multivariate analysis to identify associated factors with fecal carriage of bacteria among food handlers in prison and nonprison food establishment settings in Shewa Robit town.

Variables	Category	Bacterial colonization		COR (95% confidence interval [CI])	*p*‐Value	Adjusted odds ratio (95% CI)	*p*‐Value
		Yes	No				
Habit of wearing hair garment	Yes	36	75	Ref			
	No	67	206	1.47 (0.91–2.39)	0.11	1.49 (0.83–2.66)	0.175
Hands‐washing practice after restroom	Soap	44	85	Ref			
	Water alone	45	171	1.96 (1.2–3.21)	0.007	2 (1.16–3.45)	**0.012** *
Hand wash before food preparation	Yes	83	245	Ref			
	No	20	36	0.61 (0.33–1.11)	0.10	0.99 (0.43–2.24)	0.98
Regular medical checkups	Yes	34	55	Ref			
	No	69	226	2.025 (1.22–3.35)	0.142	2.49 (1.35–4.59)	0.003*
Food facility settings	Prison	38	68	2.68 (1.22‐5.9)	0.014	1.25 (0.51–3.06)	0.62
	Hotel	42	112	1.80 (0.83–3.88)	0.13	1.34 (0.58–3.08)	0.49
	Restaurant	13	53	01.17 (0.47–2.93)	0.72	0.36 (0.12–1.05)	0.63
	Café	10	48	Ref			
Food safety and handling training	Yes	42	70	Ref			
	No	61	211	2.07 (1.28–3.34)	0.003	2.33 (1.34–4.05)	0.002*
Fingernail trimming	Trimmed	87	262	Ref			
	Untrimmed	16	19	0.39 (0.19–0.80)	0.010	0.40 (0.16–1.00)	0.52

Abbreviation: COR, crude odds ratio.

*Significant variables at *p* < 0.05.

## 4 DISCUSSION

Foodborne illness is a leading cause of illness and mortality worldwide. Food handlers may put customers at risk of bacterial infection.^21^ Among asymptomatic food handlers, the rate of bacterial carriage in feces and hands were 102 (26.7%), and 214 (55.7%), respectively. The rate of bacterial fecal carriage was 102 (26.7%), greater than the reports in Gondar (13.2%),^22^ Jimma (6.9%),^23^ and Thailand (2.5%).^24^ Moreover, the prevalence of food‐borne pathogens among food handlers in the prison setting was 72.6% (77/106; from hand swab) and 34.9% (37/106; from fecal samples). This finding may indicates that the prisoners are at risk and more vulnerable compared to the general population for acquisition of food‐borne illness due to limited washing facilities, inadequate and clean water supplies which results poor personal hygiene, and overcrowding nature of the prisoners living in one prison houses/cells in Ethiopia.

The majority of bacteria found in the fecal samples was *E. coli* (18.5%) which is consistent with a study in Gondar that found *E. coli* to be prevalent.^25^ However, a study reported in Jimma indicated that *Salmonella* species (40%) was predominant.^23^ The prevalence of *E. coli* O157:H7 strains was 4.2%, which is lower compared to studies done in Southern Ethiopia (26%)^26^ and Nepal (10.3%).^27^ This finding was also consistent with a study conducted in Ethiopia (1.8%).^23^ In addition, the prevalence of fecal *Salmonella* species was 1.8%, which is consistent with a study reported in Southern Ethiopia (2.1%).^28^ However, the prevalence of fecal *Salmonella* species in this study was lower than studies documented in Northwest Ethiopia (4.1%),^29^ and Addis Ababa (3.5%),^5^ Sudan (4.4%),^30^ and Pakistan (9%).^31^

There was no fecal *Shigella* species identified among food handlers, which is similar with a finding in Southern Ethiopia.^28^ The hand carriage rate of bacteria was 56.2% in this study, which is higher than a study done in Debre Markos (29.5%).^32^ Our findings was also lower than studies reported in Gondar (70%),^33^ and Iran (62.2%).^34^ In this study, coagulase‐negative *staphylococci* (29.9%) was predominant followed by *S. aureus* (17.2%) and *E. coli* (6.8%). These findings are similar with a study documented in Saudi Arabia (39.9% vs. 17.5%).^35^

The hand carriage rate of *E. coli* was 6.8%, which is similar with a study in Jimma (10.9%)^36^ but lower than a study conducted in Nigeria (1.8%).^35^ The discrepancies might be due to variations of bacterial identification techniques, personal hygiene, hygiene practices during food processing and handling, and study settings. Good food safety practice is essential for the prevention and control of foodborne illness and it is a priority concern to protect the health of the community.

*E. coli* from stool samples showed highest resistance to sulfamethoxazole‐trimethoprim (60.5%), ciprofloxacin (40.8%), chloramphenicol (30.9%), which is comparable to a study done in Qatar, sulfamethoxazole (33.3%), ciprofloxacin (14.2%), and chloramphenicol (3.9%).^37^ and Morocco, reports as the highest resistance rates were shown against ampicillin and amoxicillin‐clavulanate acid (100%), and cefotaxime (60%).^38^ This study also indicated that there is a concern on the rate of *E. coli* O157:H7 strains, accounting 4.2%. *E. coli* O157:H7 strains had chloramphenicol and trimethoprim‐sulfamethoxazole resistance each 50%, which is consistency with a study done in Nigeria, where the prevalence of chloramphenicol and trimethoprim‐sulfamethoxazole resistance were 60.4% and 31.2%, respectively.^39^ This distribution of *E. coli* O157:H7 strains indicated that there is a poor food hygiene practices and its transmission to the customers, which is also supported in the associated factors analysis in this study. The hot environment of Shiwa Robit town may pique the interest of food handlers during food handling and processing, which results to poor food hygiene practices. This results that the consumers served in these food outlets are at risk of developing food‐borne illness and *E. coli* O157:H7 infections.

The results of our study revealed that the *S. aureus* isolates were resistant to trimethoprim‐sulphamethoxazole (57.6%), ciprofloxacin (49.9%), and gentamycin (49.9%). This report was significantly less than that of the study that was conducted in Debre Markos, Ethiopia, which involved ciprofloxacin (9.1%) and trimethoprim‐sulphamethoxazole (18.2%).^32^ *Pseudomonas* resistant to gentamycin (100%) and ciprofloxacin (100%) in hand swab samples which showed higher levels of resistance to both antibiotics (ciprofloxacin [33%], gentamycin [33%]) than a study carried out in Debre Markos, Ethiopia.^32^

*Salmonella species* isolated from fecal samples were highly resistant to trimethoprim‐sulfamethoxazole (57.1%), and ciprofloxacin (42.9%), which is comparable to a study conducted in Dilla, Ethiopia, where the prevalence of ampicillin (81%) and amoxicillin‐clavulanic acid (>70%) resistance was seen.^40^ Our finding was higher than a study reported in Hawassa, Ethiopia, where the prevalence of tetracycline, ampicillin, and trimethoprim‐sulfamethoxazole resistance was 80%, 60%, and 40%, respectively.^28^ The blind prescription medications in the clinical domain may be the cause of this high level antibiotic resistance. As a result, there will be no alternative medications available to treat highly drug‐resistant strains.

According to the results of the current study, *Pseudomonas* from hand swab samples exhibit 100% resistant to both gentamycin and ciprofloxacin, which is higher than the result of study in Debre Markos, Ethiopia, where both ciprofloxacin and gentamycin resistance were found to be 33%.^32^ This finding indicated that MDR bacteria accounted for 18% of the bacterial isolates. An increase in MDR could be attributed to the improper and inappropriate use of antimicrobial drugs as a result of empirical treatment and inadequate infection prevention and control measures, such as environmental cleaning and hand hygiene.

Interesting findings have been made about the association between hand washing practice with just water and bacterial colonization. Compared to hand washing with soap, the practice of hand washing after using the restroom with just water was strongly linked with bacterial colonization. This finding was supported by previous studies in both Adigrat and Arba Minch, Ethiopia.^41^, ^42^ In fact, washing hands with soap is more effective than with water alone because the surfactants in soap help to remove dirty and bacteria from the skin, and individuals prefer to wash their hands more thoroughly with soap, which helps to further eliminate germs.

Those food handlers who had an irregular medical checkups were significantly associated with bacterial colonization compared to the counterparts. A study conducted in Dilla, Ethiopia is comparable to this one.^40^ This might be due to the fact that food handlers who examined their health status regularly had a better understanding about safe food handling and processing. In addition, they also get professional advices during medical cheek‐ups which enhances their overall performance in safe food handling practices. Food handlers who didn't receive food safety and hygiene training were 2.33 times more likely to be colonize by bacteria compared to the counterparts. This finding also supported by a study conducted in Jimma, Ethiopia.^23^ Food handlers who were properly trained can take the necessary precautions to avoid malpractice during food handling and safety. A meta‐analysis study in Ethiopia supports this study which stated that food handlers with formal education, good knowledge, received training, and a positive attitude about food hygiene components, as well as regular medical checkups were significantly associated with good food hygiene practice.^43^ Therefore, food handlers should attend proper training in the basic principle of food safety and rules of personal hygiene to improve their practices during food handling. This study have some limitation such as the nature of cross‐sectional study which could not assess temporality of the cause‐effect relationships, and recall bias for some questions among participants.

## 5 CONCLUSIONS

The carriage rate of bacteria and their drug resistance among asymptomatic food handlers are a priority. *E. coli*, *S. aureus*, and CONS were the most predominant bacteria identified from food handlers, and the more risky *E. coli* O157:H7 strain was identified. The rate of MDR of bacteria was 18%. This indicates that food handlers may transmit food‐borne disease to the consumers including drug‐resistant bacteria. Hand washing practices after restroom with water alone, irregular medical checkups, and do not receive food safety and hygiene training were significantly associated with bacterial colonization. We recommend that food handlers undertake regular medical checkups, health education, and continuous food safety and hygienic practice trainings to prevent the transmission of food‐borne diseases to the customers and the community at large.

## AUTHOR CONTRIBUTIONS

**Kalu Adefrash**: Conceptualization; investigation; methodology; validation; writing—review and editing; software; formal analysis; data curation; visualization. **Bekele Sharew**: Conceptualization; investigation; methodology; validation; writing—review and editing; data curation; supervision; project administration; formal analysis. **Wubalem Amare**: Methodology; writing—review and editing; data curation; validation. **Agumas Shibabaw**: Conceptualization; investigation; writing—original draft; methodology; validation; software; formal analysis; project administration; data curation; supervision; visualization.

## CONFLICT OF INTEREST STATEMENT

The authors declare no conflict of interest.

## TRANSPARENCY STATEMENT

The lead author Agumas Shibabaw affirms that this manuscript is an honest, accurate, and transparent account of the study being reported; that no important aspects of the study have been omitted; and that any discrepancies from the study as planned (and, if relevant, registered) have been explained.

## Data Availability

The authors confirm that the data supporting the findings of this study are available within the article. If further supplementary data is required, the required data will be made available without restriction by the corresponding author.
